# Cardiac CT for personalized phenotyping in stable coronary artery disease: toward precision medicine

**DOI:** 10.1093/bjro/tzag014

**Published:** 2026-06-22

**Authors:** Joel Lenell, Kajetan Grodecki, Jacek Kwiecinski, Piotr J Slomka, Marc R Dweck, Michelle C Williams, Marc Dewey, David E Newby, Damini Dey

**Affiliations:** Biomedical Imaging Research Institute, Cedars-Sinai Medical Center, Los Angeles, CA 90048, United States; Biomedical Imaging Research Institute, Cedars-Sinai Medical Center, Los Angeles, CA 90048, United States; 1st Department of Cardiology, Medical University of Warsaw, Warsaw 02-091, Poland; Department of Interventional Cardiology and Angiology, Institute of Cardiology, Warsaw 04-628, Poland; Department of Medicine, Cedars-Sinai Medical Center, Los Angeles, CA 90048, United States; British Heart Foundation, Centre for Cardiovascular Science, University of Edinburgh, Edinburgh EH16 4TJ, United Kingdom; British Heart Foundation, Centre for Cardiovascular Science, University of Edinburgh, Edinburgh EH16 4TJ, United Kingdom; Department of Radiology, Charité - Universitätsmedizin Berlin, Corporate Member of Freie Universität Berlin and Humboldt-Universität zu Berlin, Berlin 10117, Germany; British Heart Foundation, Centre for Cardiovascular Science, University of Edinburgh, Edinburgh EH16 4TJ, United Kingdom; Biomedical Imaging Research Institute, Cedars-Sinai Medical Center, Los Angeles, CA 90048, United States

**Keywords:** precision medicine, cardiac CT angiography, plaque, myocardial infarction, chronic coronary syndrome, ischemia

## Abstract

Following technological developments and new landmark trials, the diagnostic work-up of symptomatic chronic coronary artery disease (CAD) has evolved. Clinical guidelines now favor noninvasive anatomical assessments by coronary CT angiography (CCTA) as the first-line modality to evaluate CAD in the majority of patients with chest pain. This shift from ischemia testing to stenosis and plaque characterization has resulted in the development of new imaging biomarkers reflecting a variety of coronary plaque features, many of which have proven to be important clinical risk markers. Consequently, there has been a transition from qualitative to semi-quantitative and fully quantitative plaque acquisitions over the entire coronary tree. With the integration of artificial intelligence, novel software enables rapid quantitative acquisitions of plaque components, making them feasible for use in clinical practice. CCTA has also enabled identification of precursor features associated with plaque development such as peri-coronary artery adipose tissue attenuation and epicardial adipose tissue volume. This review provides an overview of CCTA derived plaque features in CAD and associated imaging biomarkers of risk to highlight their potential applications in precision phenotyping and individualized management decisions. It further outlines anticipated future developments that may enable widespread clinical adoption of these novel imaging biomarkers.

## Developments in clinical management of chronic coronary artery disease

For many clinicians, coronary artery disease (CAD) management was based on the belief that severe epicardial coronary artery stenoses were the sole drivers of anginal symptoms and adverse outcomes. This view is at odds with new data that have challenged these ideas.^1^ The mortality benefit from revascularization in subjects with obstructive CAD has been in question since the COURAGE trial back in 2007 and lack of survival benefit was later confirmed by the ISCHEMIA trial in 2020.^2^^,^^3^ Furthermore, the ORBITA trial, a sham-controlled trial of percutaneous coronary intervention (PCI), reported no improvement in anginal symptom relief from PCI beyond medical treatment in subjects with single vessel disease.^4^ In the ORBITA II trial, patients who stopped their anti-anginal medication 2 weeks in advance of PCI demonstrated a symptomatic improvement in the PCI arm at 12 weeks but the magnitude of this benefit was similar to that of established anti-anginal pharmacotherapies.^5^

Although only studying selected populations, together, these trials established a more nuanced approach to management of the chronic coronary syndrome (CCS) where patients and clinicians have greater equipoise between the choice of either medical treatment or invasive PCI to relieve anginal symptoms caused by obstructive CAD. Given that medical treatment and PCI appeared to offer similar symptom control, epicardial stenoses cannot be considered the sole drivers of anginal symptoms.

In recent international guidelines, there is an emphasis on noninvasive imaging, and particularly coronary CT angiography (CCTA), in the diagnostic work-up to guide management of patients with chest pain.^6^ CCTA offers important information on the anatomical state and contributes prognostic information derived from a range of novel imaging biomarkers which have the potential to guide patient phenotyping and the tailoring of personalized patient management.

Precision medicine has gained increased interest in the last decades and refers to the tailoring of patient management based on unique individual features.^7^ The personalization of disease prevention and treatment has been made possible following readily available metrics for individualized phenotyping. This review focuses on the current and future use of cardiac imaging by CT in the evaluation of CAD and addresses possible future applications of artificial intelligence (AI) derived metrics for precision phenotyping to enable personalized management.

## Plaque and stenosis assessment

### Qualitative assessments of stenosis severity

Coronary CT angiography is established as a first-line modality to evaluate symptomatic CAD owing to excellent sensitivity and specificity in identifying coronary plaque.^8^ Furthermore, CCTA can reliably exclude hemodynamically significant disease^6^ and patients who first undergo a CCTA have lower interventional complication rates compared to those who go straight to an invasive angiogram, which emphasizes the benefit of CCTA as the first modality in chest-pain work-up.^9^ CCTA has also proven valuable in guidance of pharmacological interventions as demonstrated in the 10-year follow-up of the SCOT-HEART trial wherein patients with CCTA confirmed CAD were assigned to preventive treatment, including statins and aspirin, leading to a lower incidence of myocardial infarctions (MIs) and cardiovascular death when compared to standard care.^10^

It was recently reported that individuals undergoing CCTA with findings of CAD are more prone to adhere to prescribed therapies and to adopt a healthier lifestyle; leading to lower cholesterol concentrations and lower blood pressure than in those receiving lifestyle advice without a CCTA.^11^ These findings support the advantage of using patient specific CCTA findings in clinical communication to improve compliance with recommended treatments.

**Figure 1:**
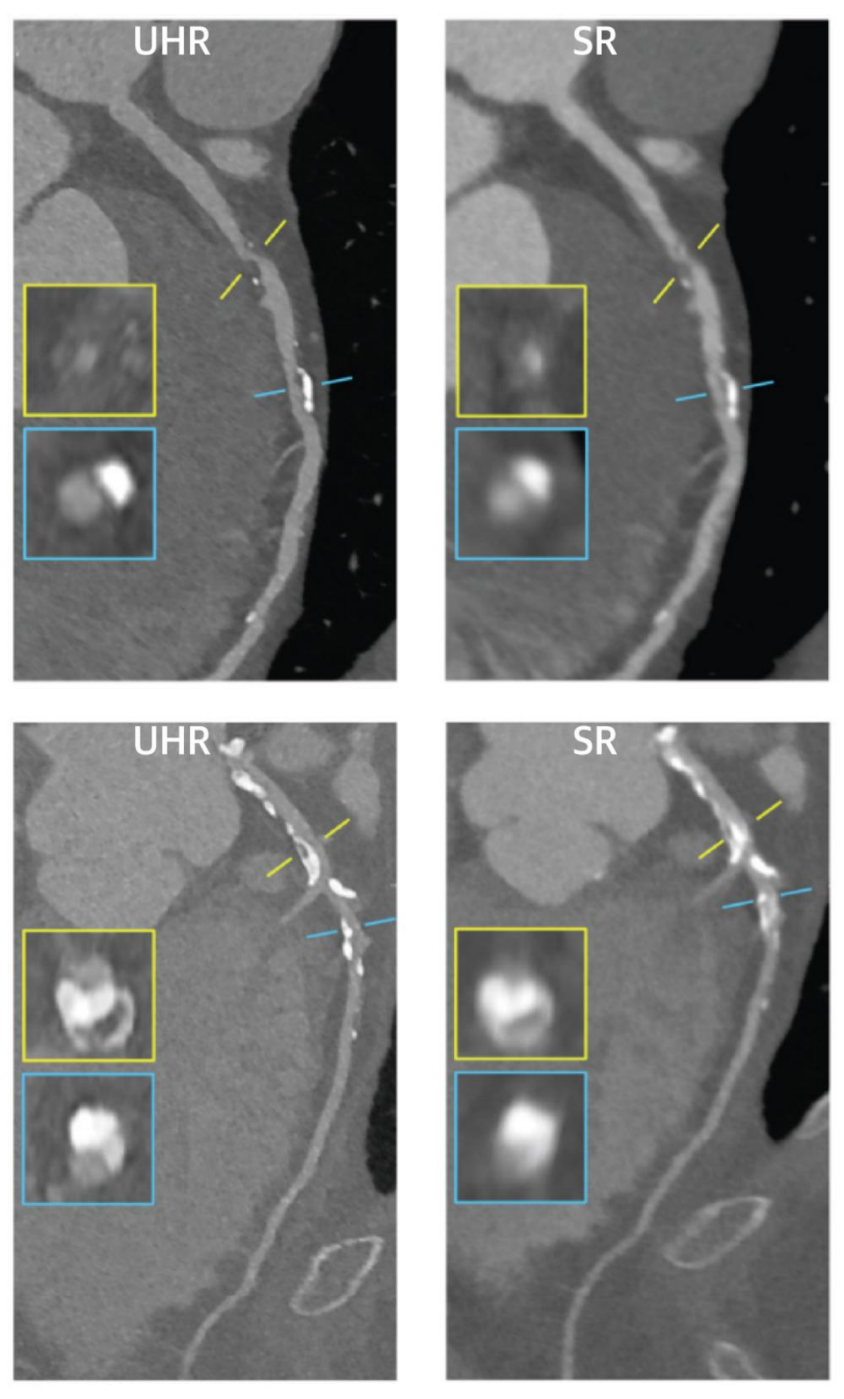
The left anterior descending artery in two patients (upper and lower) demonstrating differences in image readability between ultra-high resolution vs standard resolution photon counting detector CT scans. Ultra-high resolution (UHR); standard resolution (SR). Adapted and reproduced from Kotronias et al.^13^ under a creative commons license.

With the advent of ultra-high resolution (UHR) photon counting detector (PCD) scanners in clinical practice, previous issues associated with the partial volume effect and blooming have been reduced, allowing for reliable grading of stenoses (Figure 1).^12^^,^^13^ A recently published paper reported an accuracy in significant stenosis adjudication from UHR PCD CCTA per patient at 88% (sensitivity 96% and specificity 84%) with invasive angiogram as reference, reflecting an important improvement to standard energy integrating detector (EID) scanners.^14^ Another study reported a positive predictive value for revascularization at 83.3% vs 63.0% (*P* = .002) by PCD CT vs EID CT.^15^

Grading of stenosis severity may help to determine a lesion’s contribution to anginal symptoms but the severity is less specific in prediction of future events caused by an individual plaque.^16^ Instead, characterization of plaque composition may provide a more specific estimate of the risk of an impending infarction.

### Qualitative plaque assessments

Visually assessed high-risk coronary artery plaque features, also known as vulnerable plaque features, include positive remodeling (PR) defined as an increase in coronary caliber of at least 10% at the site of an atherosclerotic plaque (found in ∼1.0% of lesions), low attenuation plaque (LAP), <30 Hounsfield Units (found in ∼0.8%), napkin ring sign (found in ∼0.4%), and spotty calcification defined as calcifications <3 mm (found in ∼13%).^17^^,^^18^ The napkin ring sign is a surrogate marker for a thin cap fibroatheroma with a necrotic core and overlaps with PR and LAP in roughly 80% of cases.^17^ The napkin ring sign alone has been reported to offer a sensitivity of 42% and a specificity of 97% in prediction of future acute coronary syndrome (ACS).^17^ PR is associated with a higher degree of lipid accumulation and a lower burden of calcium in the overall coronary tree.^19^ Spotty calcification, being the most prevalent adverse plaque feature, is less specific and consistently presents with a lower hazard ratio than the other markers in multivariable models assessing time to ACS.^20^ By convention, at least 2 of these high-risk features are needed for a plaque to be labeled as a high-risk plaque (HRP).^21^

### Semi-quantitative plaque assessments

In the SCOT-HEART trial, HRP features did not emerge as independent prognostic markers of death or MI when analyzed together with the semi-quantitative Coronary Artery Calcium (CAC) score.^22^ CAC is calculated from a standardized non-contrast scan protocol with calcium defined as an area of 1 mm^2^ with an attenuation greater than 130 Hounsfield units (HU). The final Agatston score is derived from the total area of calcium multiplied by a density factor and could be considered a surrogate marker of overall plaque burden (both calcified and non-calcified). More plaque is associated with higher event rates; hence the CAC score is a valuable prognostic marker but naturally offers no guidance in tailoring of invasive interventions.

The CAC score has proven useful in guiding statin treatment to reduce HRP in the CAUGHT-CAD trial, demonstrating a feasible imaging informed pharmacotherapeutic strategy.^23^ However, follow-up data from the SCOT-HEART trial showed that a substantial minority (10%) of MIs occurred in subjects with a baseline CAC of 0 which demonstrates a weakness in CAC guided management among symptomatic patients.^24^

Another semi-quantitative plaque assessment tool is the updated CAD-RADS 2.0 reporting system that has been adopted in recent years.^25^ CAD-RADS 2.0 integrates information on stenosis severity with a classification of overall plaque burden derived from CAC and/or the segment involvement score (SIS). However, future versions of CAD-RADS are likely to inform on total plaque burden derived from quantitative measurements as these applications are adopted in clinical practice.^26^

### Quantitative plaque assessments

Semi-quantitative metrics like CAC scoring have been made readily available in the clinic by AI-enabled software. However, novel applications can accurately quantify not only calcified plaque (CP) but also fibrotic and fatty plaque components in good agreement with invasive intravascular ultrasound.^27^^,^^28^ Furthermore, overall non-calcified plaque (NCP) burden is a superior marker of total plaque burden and of risk for future MI compared to CAC.^16^

**Figure 2:**
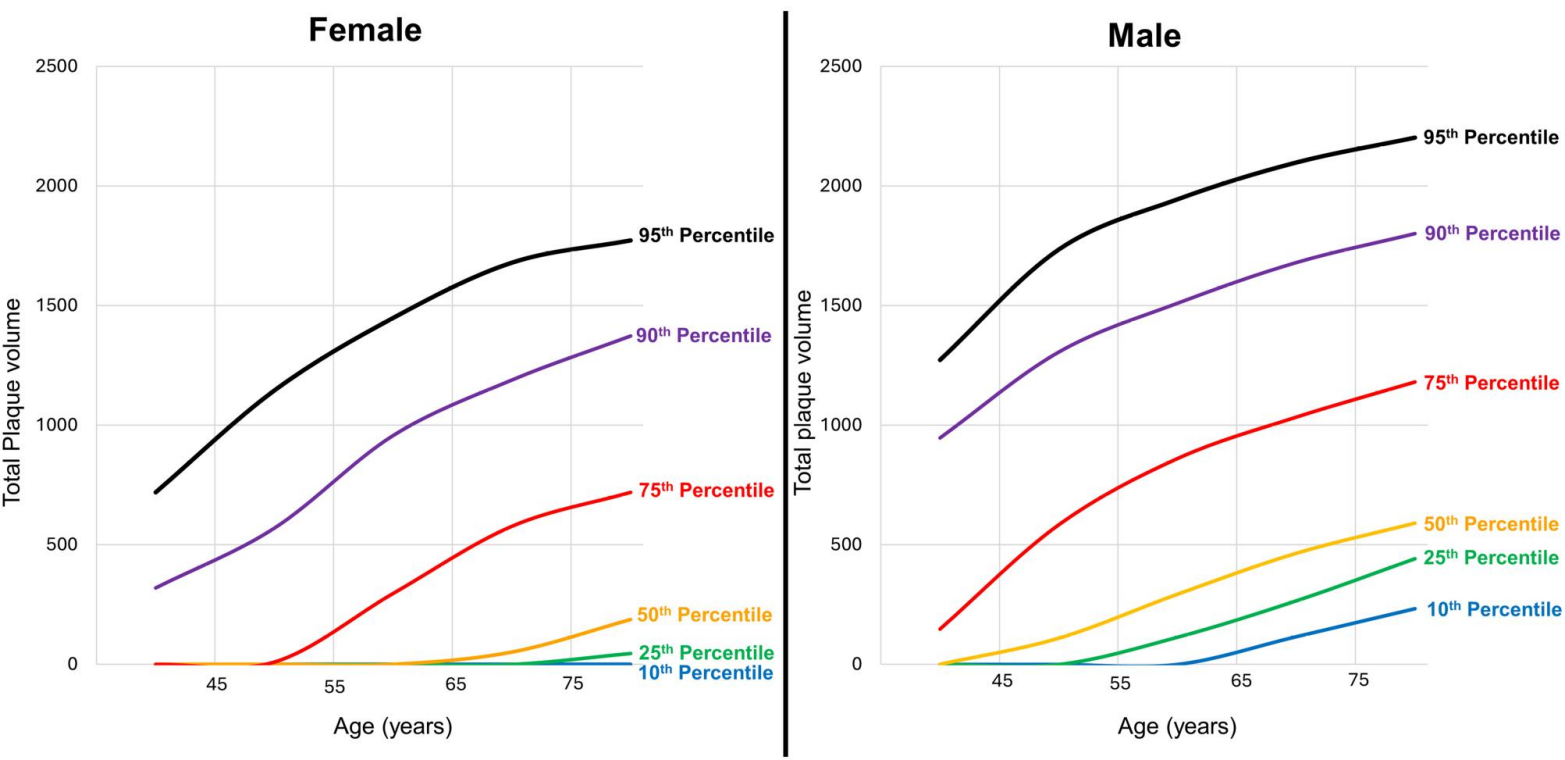
Age and gender stratified percentiles for total plaque volume to be used for individualized risk stratification and patient communication. Reproduced with permission from Miller et al.^29^

Tailored reference values are imperative for precision in individual phenotyping and downstream clinical decision making. Recent advances in quantitative plaque imaging involve the development of age and sex stratified distributions of total plaque volume in patients undergoing CCTA for CAD work-up. These data are now available, much like the percentile curves routinely presented alongside a CAC score, which will facilitate reliable clinical decisions and patient communication when addressing disease burden (Figure 2).^29^

Plaque volumes are typically assessed in 3 main components: CP, NCP, and low-density NCP with their sum representing the total plaque volume. Low-density NCP volume serves as a quantitative measure of LAP and PR can be quantified by the remodeling index, allowing for refined characterization beyond the qualitative HRP features.

The prognostic value of quantitative plaque features has been validated clinically both in patients presenting with stable and acute chest pain.^30^ Those with ACS more frequently had a lower proportion of dense calcium and a higher proportion of fibro-fatty or necrotic core tissue as adjudicated by plaque attenuation features than those presenting without ACS.^31^ It should also be noted that higher volumes of high-density plaque, typically termed “1K Plaque” with reference to plaque of ≥1000 HU, are associated with lower risk of future ACS.^32^ There are recently developed and validated applications derived from deep-learning convolutional neural networks that allow for automated plaque volume quantification across all these components, effectively replacing manual input from the reading physician.^33^

**Figure 3:**
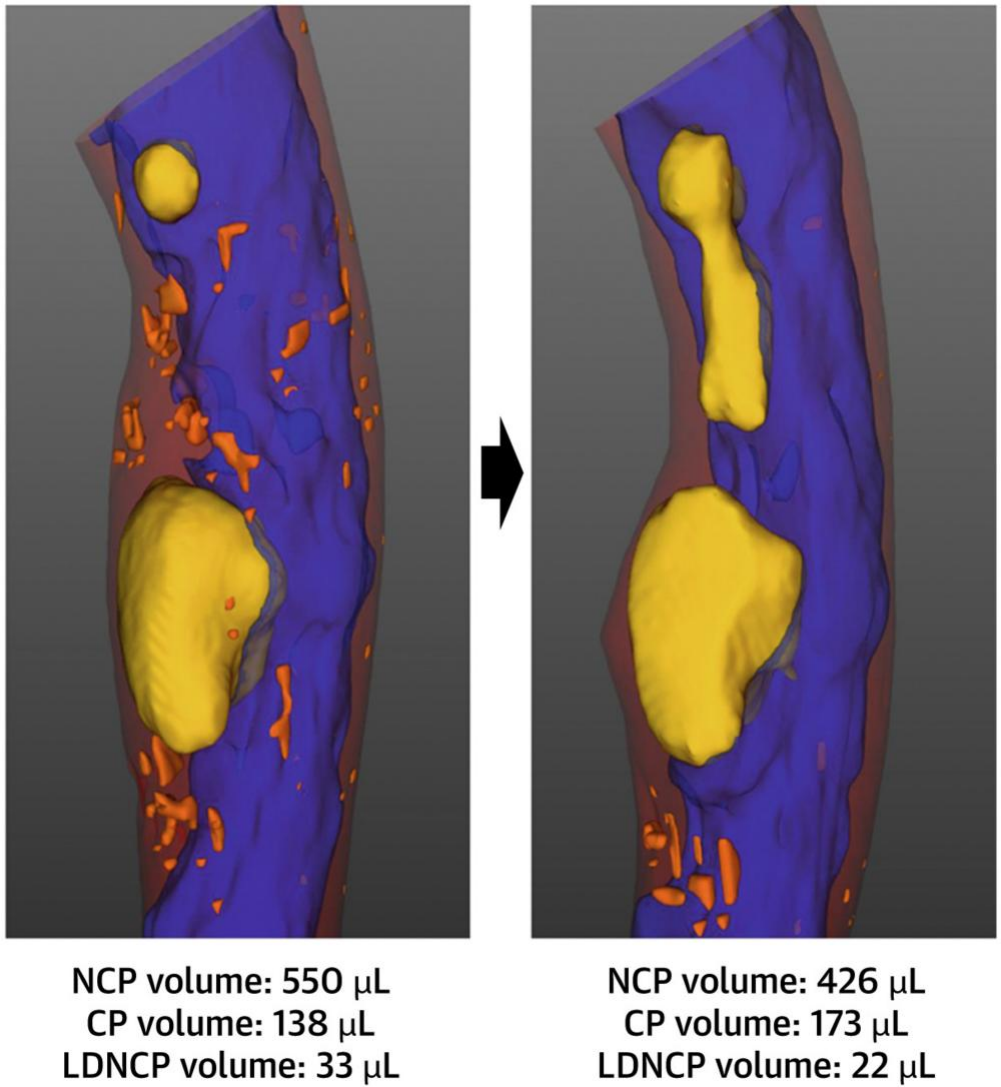
Example of serial CCTA under Evolocumab treatment with a reduction in non-calcified and low-density non-calcified plaque, accompanied by an increase in calcified plaque. Calcified plaque (yellow), non-calcified plaque (red); low-attenuation features (orange). Reproduced with permission from Han et al.^36^

Quantitative plaque acquisition is also key to following CAD longitudinally by serial CCTA scanning and enables monitoring of treatment response. Statin treatment has been shown to promote an increase in CP volume at the expense of NCP, revealing a pathophysiological mechanism for the favorable effects observed in clinical outcomes.^34^^,^^35^ Similar findings have been reported under treatment with Evolocumab (Figure 3).^36^ An upcoming analysis of change in plaque characteristics is expected from the SCAPIS cohort,^37^ having enrolled approximately 15 000 participants for a repeat CCTA in the SCAPIS 2 study (NCT06679777). Another awaited serial scan study is the WARRIOR Ancillary Serial Imaging study (NCT03417388) examining changes in plaque composition among women with suspected Ischemia and non-obstructive CAD (INOCA). Identification of early favorable plaque changes under ongoing treatment could potentially further motivate patients to adhere to medical treatment, ultimately leading to improved clinical outcomes.

## Ischemia testing

### Myocardial perfusion

Myocardial perfusion imaging by cardiac CT has witnessed reluctant adoption over the years with critics raising concerns related to radiation and contrast dosage from repeated scanning.^38^ However, with advances in radiation reduction techniques it is becoming an increasingly more feasible option. The use of deep learning-based reconstructions has further improved image quality and enabled lower radiation doses.^39^ It should also be noted that the increasing availability of regadenosone as a stress agent has increased patient tolerability and clinical feasibility of pharmacological stress perfusion scans.^40^

Modern PCD scanners have been reported to allow for accurate static perfusion assessments validated by stress CMR or invasive angiography^41^ with an acceptable mean dose length product at 386 ± 74 mGy cm. These dual source scanners with spectral data also enable quantification of iodine density in the myocardium which helps to single out regional differences in perfusion.

Dynamic CT perfusion involves serial acquisitions at short intervals during first-pass perfusion of the myocardium.^42^ This allows for both a qualitative visual assessment of perfusion and also a quantified absolute estimate of myocardial blood flow (MBF) that enables diagnosing of microvascular disease. The reliability of perfusion defects from epicardial stenoses by CT perfusion is markedly impaired in the presence of microvascular dysfunction which underscores the superiority of quantitative MBF assessments.^43^ However, novel software allows for the assessment of hemodynamic stenosis severity from a standard CCTA examinations which may limit the clinical need for perfusion imaging in the evaluation of epicardial stenoses.

### CT fractional flow reserve

CT fractional flow reserve (FFR) (FFR_CT_) can be derived by computational fluid dynamics (CFD) to estimate hemodynamically obstructive coronary stenoses.^44^ A recent meta-analysis of 43 studies, with a total of 5236 patients reported its performance in prediction of significant stenoses (invasive FFR ≤0.80) with an accuracy of 82.2%, a sensitivity of 80.9% and a specificity of 83.1%.^45^ The authors concluded that FFR_CT_ can rule out hemodynamically significant CAD in cases with an FFR_CT_ >0.90, with an accuracy of 90%. To reliably rule in hemodynamically obstructive CAD with an accuracy of 90%, the authors reported an FFR_CT_ threshold at <0.49, suggesting that FFR_CT_ may be used as a gatekeeper to invasive assessments. There is trial evidence that FFR_CT_ indeed may be used to reduce the number of invasive angiographies although there is yet no trial data to support improved outcomes from FFR_CT_ informed PCI.^46^^,^^47^

## Extra-coronary imaging biomarkers

### Perivascular fat attenuation

Attenuation in peri-coronary adipose tissue (PCAT) has been proposed as a measure of inflammatory activity and is associated with the presence of high-risk plaques.^48^^,^^49^ PCAT attenuation normally exhibits an attenuation range of −190 to −30 HU/voxel depending on its fat and water composition.^50^ Increased attenuation, with a cut off around −75 to −70 HU, is thought to reflect higher water content which is consistent with an active inflammatory response.^48^^,^^51^^,^^52^ This inflammation is associated with progression of NCP burden, suggesting PCAT as a useful imaging biomarker for identification of high-risk individuals. Findings from the SCOT-HEART trial and the ORFAN cohort have further confirmed the prognostic value of PCAT attenuation with respect to future MI and cardiovascular death which emphasizes its clinical relevance.^53^^,^^54^

The PCAT attenuation of culprit plaques has been demonstrated to exhibit significantly higher values than in previously stented obstructive lesions.^48^ This could translate to more precise adjudications of potential culprit plaques in need of intervention. Indeed, treatment with anti-inflammatory agents or statins has rendered a decrease in PCAT attenuation across different cohorts which indicates that it may be a modifiable biomarker suitable for clinical surveillance.^55^^,^^56^

### Epicardial adipose tissue

**Figure 4:**
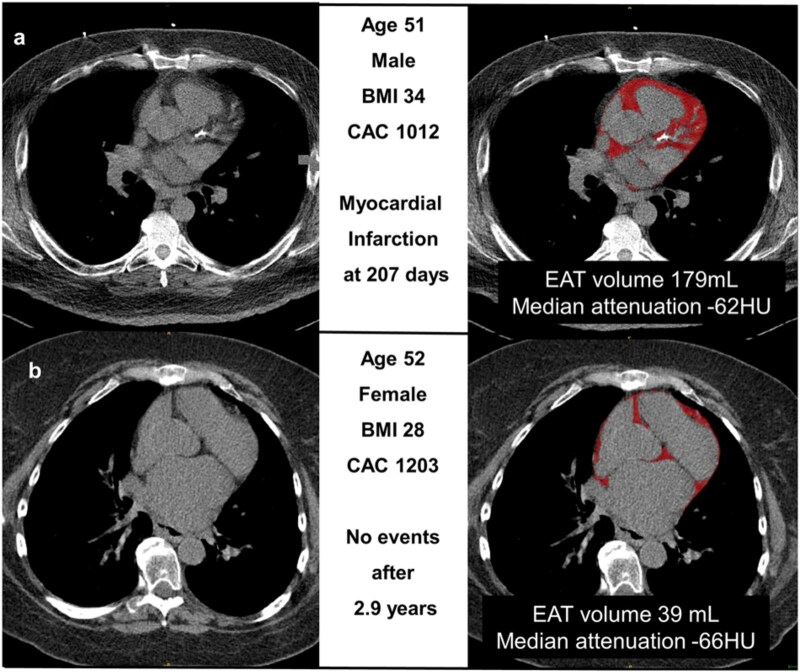
Example of automatic segmentations of epicardial adipose tissue volume from non-gated non-contrast chest scans. (a) Case example of a male patient with high epicardial adipose tissue volume. (b) Case example of a female patient with normal epicardial adipose tissue volume. Reproduced with permission from Miller et al.^62^

There is adipose tissue not only encasing the coronary arteries, but the entire myocardium (Figure 4). The total volume of epicardial adipose tissue (EAT), now eligible for automatic quantification, is associated with a MACE composite of MI, invasive revascularization, and cardiac death.^57–59^ Furthermore, local EAT volume in each coronary vascular territory is independently associated with levels of coronary artery calcium in the adjacent vessel.^57^ The coronary arteries share microvasculature with the metabolically active EAT which suggests a causative interplay between EAT inflammation and CAD.^60^ Indeed, not only EAT volume, but also EAT attenuation, a surrogate marker for inflammatory activity, has been demonstrated to predict the calcium density score in nearby coronary arteries.^57^ Given the hypothesized interaction between inflammatory EAT and the CAD progression, EAT volume and attenuation could evolve into interesting endpoints in future intervention studies.^61^

### Body composition

Deep-learning algorithms have recently enabled automated CT-based acquisitions of body composition.^63^ These data include quantitative information on tissues like skeletal muscle, visceral fat, and subcutaneous fat. A recently described model derived from attenuation corrected perfusion scans has extended the acquisitions to include volume and attenuation of bone and skeletal muscle along with subcutaneous, intramuscular, visceral, and epicardial adipose tissue.^64^ The investigators found that bone attenuation was closely related to survival with higher attenuation associated with lower risk of death. Also, muscle volume carried important prognostic information. This comprehensive body composition assessment may allow for precise phenotyping and risk assessments in routine scanning, providing information on wider aspects of the cardiometabolic disease burden beyond the coronary arteries.

## Artificial intelligence

### AI-enabled CCTA analysis

The integration of AI in imaging software applications has vastly reshaped the way coronary examinations can be assessed. By benefitting from deep-learning algorithms, Lin et al reported a reduction in the time required to assess total plaque volume in 1 patient from 25 min to 6 s.^33^ This kind of time saving applications are key to enable clinical adoption of novel imaging biomarkers. Even more importantly, plaque volumes derived from this automated deep-learning model outperformed the 10-year clinical risk score in prediction of future MI. Another group recently reported the superior performance of AI-derived stenosis severity grading vs level 3 and level 2 clinical readers with invasive angiography as reference, emphasizing the excellent accuracy achievable by AI models.^65^

An important application of AI is the comprehensive integration of multiple CCTA derived plaque features to aid risk stratification and clinical decision making. One such example is the AI-ischemia risk score that was developed and validated for prediction of hemodynamically obstructive coronary artery stenoses (defined by invasive FFR ≤0.80%).^66^ The AI-ischemia score integrates quantitative plaque features together with age and gender in a model built with a boosted ensemble algorithm to yield accurate predictions. This kind of machine-learning tools are particularly useful to extract prognostic information from several different imaging features into a single predictive model.

### Radiomics

In the realm of precision medicine, large prognostic datasets are often derived from so called “OMICS”. The term OMICS comes from the Greek word “ome,” meaning “whole” or “group,” and is widely used in characterization of comprehensive sets of biological data such as genomics, transcriptomics, proteomics, and metabolomics. All of these may contribute personalized information on patient diagnosis, prognosis, and expected treatment response. The term *radiomics* has been adopted with reference to the comprehensive tissue characterization enabled by medical imaging.

Radiomic features from coronary plaque have demonstrated excellent accuracy in identifying high-risk plaques, and more importantly, they are highly accurate at predicting culprit lesions independently of qualitative plaque features.^67–69^ Furthermore, outcome prediction using texture analysis of PCAT can improve prediction of MACE in addition to traditional imaging risk factors, such as CAC, stenosis grade, and HRP features.^52^ Radiomic phenotyping has also revealed distinct PCAT features in patients with stable CAD that allows for prediction of future MI with higher accuracy as compared to solely attenuation-based models.^70^

Shu et al extracted radiomic features from the myocardium in CCTA images to identify ischemia as determined by SPECT perfusion imaging.^71^ Their training, test, and validation sets demonstrated an accuracy of 0.839, 0.832, and 0.816, respectively. Other studied applications of radiomics in heart disease include characterization of left ventricular remodeling in hypertension and the characterization of cardiac masses.^72–74^ The abundance of information extractable from regular clinical examinations and their strong associations to outcome measurements found in observational data are highly encouraging in a fast moving and evolving field.

### Barriers to clinical adoption

Technical challenges in quantitative CCTA imaging are partially related to difficulties in standardization of image acquisitions, affecting both interpretability and reproducibility of results. In clinical practice, mode of scanning and image processing is typically tailored after patient characteristics, such as body size, heart rate, blood pressure, and coronary disease burden, by adjustment of scanner acquisition settings, pretreatment with beta-blockers/nitroglycerin, contrast dose, and choice of reconstruction kernel.

Lower tube voltage is known to increase calcium blooming, leading to higher volumes of measured CP and lower volumes of NCP components, emphasizing the importance of consistency in scanning protocols across serial scans in individual patients.^75^ Contrast density affects plaque assessments with higher density contrast media in the coronary arteries, typically leading to an increased estimate of CP volume.^75^ It has also been reported that NCP attenuation varies with contrast density, increasing with higher lumen opacification and reducing with lower opacification, potentially affecting the adjudication of NCP subcomponents.^76^

Some software applications for quantitative analysis apply scan-specific attenuation thresholds for plaque assessments rather than fixed thresholds, thereby adjusting for tube voltage and contrast density differences but several vendors still do not offer this feature. Despite society-initiated efforts to standardize plaque quantifications, further validation is needed, particularly across different scanners and vendors.

On the patient level, changes in body size may affect attenuation in serial scans, serving as a potential confounding factor when assessing change in plaque volumes. Other acquisition related factors that may affect reliability in plaque assessments include heart rate and administration of nitroglycerin at the time of scanning. Nitroglycerin serves to dilate the coronaries and may help to delineate plaque in smaller vessels.^77^ Plaque metrics indexed to the overall volume of the coronary tree, often referred to as plaque burden or percent atheroma volume, are likely to be particularly sensitive to the nitroglycerin effect as it may increase the assessable volume of the coronary tree.

Given the reproducibility challenges in plaque imaging, extraction of radiomic features from serial imaging suffer from the same susceptibility to changes in image acquisition and processing modes as quantifications of plaque, with particularly poor reproducibility across different scanners.^78^ However, there are initiatives to tackle this issue. A recently introduced method for harmonizing CT data from different constructs has demonstrated enhanced consistency in radiomic features across multi-energy CT data with different spectral reconstructions.^79^

## Ethical considerations

### Generation of AI models

To derive and validate AI models for precision phenotyping, large imaging databases are required. This often implies using patient data retrospectively supported by written informed consent acquired before *post hoc* studies, with the purpose of training and validating new AI models, were defined. This use of a general consent routinely gets Institutional Review Board (IRB) and Ethics Committee approval but the fact that patients often do not have an opportunity to decide on participation in *post hoc* studies may constitute an ethical dilemma and emphasizes the responsibility of researchers to adhere to established conventions, particularly the Declaration of Helsinki.^80^ Selection in patient enrollment will also lead to algorithmic bias wherein the model learns to derive imaging biomarkers from the studied population, which may not be generalizable to other populations, calling for validations in more diverse groups of people.

### Accessibility and cost implications

Functional ischemia testing has long been the dominant method in assessments of suspected obstructive CAD. Following more recent evidence showing improved outcomes from CCTA-guided management, guidelines have changed, favoring anatomical imaging as first-line modality. However, this shift comes with accessibility challenges, both due to the increasing demand for CT exams and because of competence shortage for exam interpretation.^81^ To compensate for insufficient reader training, it is possible that automated AI software may come to offer a feasible alternative for reliable risk assessments.^82^^,^^83^ A recent paper, investigating cost effectiveness of AI-enhanced quantitative CCTA risk stratification (based on PCAT attenuation, plaque burden, and clinical risk factors) vs standard care with stenosis severity guided management, concluded that the AI risk score was cost-effective. AI-model guided management was cost-effective also when compared to full implementation of the NICE guidelines for cardiovascular disease risk assessment and management, with reported incremental cost-effectiveness ratios (ICERs; the ratio of incremental costs to incremental QALYs) under £3500 per QALY for a wide range of prices for the AI-enhanced algorithm.^82^ These costs may be covered in more resourceful medical systems but it should be acknowledged that many patients around the globe will not have access to these emerging technologies at their current pricing.

It may also be difficult to widely increase scanner availability. Among 16 studied countries within the Organization for Economic Co-operation and Development (OECD), number of CT scanners per million inhabitants had increased over the last decade, as did the number of performed scans per scanner, suggesting increased availability.^83^ However, despite an absolute increase in availability among most of the studied countries, reported curves reflecting availability over time appears relatively flat, with large differences within OECD, and it is likely that several non-OECD regions have substantially lower scanner availability than those studied.

### Radiation

As CCTA has gained increased adoption in clinical practice over the last decades there is also a rising awareness of its associated radiation doses potentially causing harm. There is consensus to scan patients according to the ALARA (as low as reasonably achievable) principles, meaning that radiation is to be kept as low as possible while still allowing for a diagnostic quality exam.^84^ Efforts to reduce radiation doses have proven successful with reductions in kVp and tube current, usually accounting for the greatest reductions in radiation dose.^85^ Other radiation saving methods include high-pitch scanning, a preference for prospective triggering, and tube current modulation in retrospective spiral acquisitions.

## Translation to precision medicine

At present, the European Society of Cardiology guidelines endorse CCTA for qualitative and semi-quantitative anatomical assessments.^6^ For fully quantitative biomarkers to be confidently adopted as clinical management-directing metrics, clear thresholds are needed to facilitate management decisions.^86^ Clinical outcome trials are expensive, which typically limits the choice of phenotyping metrics to established, validated, and already adopted biomarkers. The single most used metric to guide randomized trials in cardiology is left ventricular ejection fraction (LVEF) even though this marker only is informative when myocardial injury has already occurred.^6^ Hence, there is an urgent need for prognostic markers that can risk stratify for future adverse clinical events and CCTA is uniquely well positioned in this respect.

A second-best option in evaluation of new biomarkers is the utilization of *post hoc* analyses in randomized trials to identify biomarkers that help single out phenotypes of particular benefit from the treatment intervention. To enable these analyses, it is imperative that biobanks of data for phenotyping are acquired before randomization. This has long been acknowledged within the oncologic field but has yet to expand widely within cardiology.

The SCOT-HEART 2 trial is one such ongoing landmark trial randomizing at least 6000 asymptomatic participants with 1 or more risk factors of CAD to CCTA-guided medical treatment or standard care and is expected to finish 5-year follow-up in 2030.^87^ Another ongoing trial is the TRANSFORM trial, investigating the effect of a CAD staging-system based treatment strategy vs risk-factor based care in asymptomatic patients with diabetes, prediabetes, or metabolic syndrome (NCT06112418). The findings of these trials will likely shed definitive light on the clinical importance of CCTA in guiding clinical management.

Based on current evidence, an overview of some of the most promising opportunistic quantitative CCTA biomarkers in cardiovascular disease is reported in Table 1, describing how they may be implemented to improve precision phenotyping and ultimately guide clinical management.

**Table 1:** Quantitative CCTA imaging biomarkers: current evidence, incremental value, and potential clinical impact.

Quantitative biomarker (CCTA)	What it captures	Current evidence state	How it can potentially alter clinical decision pathways
Total plaque volume	Patient level global atherosclerotic plaque volume	Consistent observational prognostic associations. Expert consensus supports standardized quantitative plaque assessments with percentile guided medical management	Reclassify non-obstructive CAD into higher-risk phenotypes which may be used to guide intensified prevention (high-intensity statin, tighter LDL targets), earlier follow-up imaging/monitoring, and stronger lifestyle/pharmacotherapy adherence communication
CP volume	Extent of calcified atherosclerosis on CCTA	Observational. Evidence base overlaps with CAC literature	May serve as a surrogate marker of total plaque and may as such be used to guide management in a similar manner as total plaque volume
NCP volume	Total noncalcified plaque volume (lipid/fibrous components)	Observational prognostic evidence showing improved risk stratification from NCP volume beyond stenosis grade	Supports earlier preventive therapy initiation (even with CAC = 0). Can shift “reassurance” toward “treat and track” when symptoms are atypical but plaque phenotype is high-risk. Can be used to guide intensity of pharmacological and lifestyle interventions
LD-NCP (LAP) volume	Low-attenuation, lipid-rich plaque component (often treated as surrogate for vulnerable plaque)	Strong prognostic signal in stable chest pain cohorts. In SCOT-HEART analyses, low-attenuation plaque burden was a particularly strong predictor of MI	Escalate prevention (high-intensity statin ± additional lipid-lowering depending on risk) and consider closer surveillance
PCAT attenuation	Imaging marker linked to local coronary inflammation	Large cohorts show associations with plaque progression and outcomes (myocardial infarction), but incremental value is heterogeneous across studies	Could help identify an inflammatory phenotype for intensified prevention and closer follow-up. At present, use is best framed as adjunctive rather than stand-alone decision trigger
FFR-CT	Computational fluid-dynamics–derived fractional flow reserve from CCTA. Lesion-specific ischemic significance (FFR ≤0.80)	Validated against invasive FFR. Multiple management trials show reduced unnecessary invasive angiography and safe deferral of revascularization	Guides revascularization vs medical therapy: patients with anatomically moderate lesions but normal FFR-CT can be safely treated medically, while those with functionally significant disease are referred for ICA
Epicardial adipose tissue volume	Total volume of fat within the pericardial sac, quantified on non-contrast or contrast CT. Reflects systemic and local cardiometabolic risk	Large observational and mechanistic literature links high EAT to CAD presence, plaque burden, adverse remodeling, atrial fibrillation, and outcomes. Less lesion-specific than PCAT but more reproducible and standardized	Supports cardiometabolic risk phenotyping: high EAT identifies patients who may benefit most from aggressive lifestyle, weight loss, GLP-1RA, SGLT2 inhibitors, and lipid lowering. In CCTA-negative or nonobstructive CAD patients, high EAT may justify earlier preventive pharmacotherapy and closer follow-up although more data is needed to define treatment thresholds

Abbreviations: CAC = coronary artery calcium; CAD = coronary artery disease; CCTA = coronary CT angiography; CMR = cardiovascular MRI; CP = calcified plaque; FFR-CT = fractional low reserve derived from coronary CT angiography; EAT = epicardial adipose tissue; FFR = fractional flow reserve; GLP-1RA = glucagon-like peptide-1 receptor agonist; ICA = invasive coronary angiograph; LD-NCP = low-density non-calcified plaque; LDL-C = low-density lipoprotein cholesterol; MI = myocardial infarction; NCP = non-calcified plaque; plaque component lacking calcium, including fibrous and lipid-rich tissue; PCAT = peri-coronary adipose tissue; PCI = percutaneous coronary intervention; SCOT-HEART = Scottish Computed Tomography of the HEART trial; SGLT2 = sodium-glucose cotransporter-2 inhibitor.

Presence of any plaque, determined by quantitative methods, is suggested to mandate initiation of statin treatment, and volumes of total plaque above the 70th age and sex adjusted centile to mandate high-intensity statin treatment (typically Rosuvastatin at least 20 mg daily or Atorvastatin at least 40 mg daily).^88^ NCP volume, often representing a substantial proportion of the total plaque volume and sometimes the entire plaque volume, captures risk not identified by CAC and can thereby guide initiation of statin therapy and lifestyle interventions also in those with no coronary calcifications. LD-NCP, reflecting plaque instability, may guide more intensive lipid lowering therapy based on observational data with a potential threshold of LD-NCP burden >4% to single out patients at particularly high risk.^16^

Peri-coronary adipose tissue attenuation greater than −70.5 HU in the proximal segment of the RCA have been suggested to identify high-risk individuals that could benefit from intensified treatment and novel anti-inflammatory medication.^52^ FFR-CT has the potential to guide selection of patients eligible for invasive assessment with a suggested threshold at ≤0.80 for invasive coronary angiography evaluation. This approach has trial validation supporting reduced downstream invasive work-up.^46^^,^^47^

## Outlooks for the future

Cardiac CT is the cornerstone in the current paradigm of CCS work-up and management, mainly attributable to its outstanding image resolution allowing for reliable anatomical assessments.

Qualitative and quantitative plaque analyses have revealed landmark insights into risks related to plaque composition. In 2024, the open-label PREVENT trial reported improved MACE outcomes following a preventive PCI strategy guided by invasively assessed high-risk coronary plaque features in non-obstructive stenoses.^89^ These results were substantially driven by target-vessel revascularization, however, with favorable directional consistency for each component of the MACE outcome. Clinically available PCD scanners with UHR reconstructions now have an isometric spatial resolution of 200 µm, which is getting closer to the 20-40 MHz IVUS resolution,^90^^,^^91^ making it increasingly feasible to investigate whether noninvasive CT-imaging of high-risk plaques could guide patient selection for PCI with favorable outcomes.

Another advancement, likely not far away, will come from the abundance of prognostic imaging biomarkers derivable from AI-enabled applications that will make way for the development of accurate risk prediction tools to guide future pharmacological therapy. We are likely to see fully automated readings of cardiac examinations, including the extraction of radiomic data, for individual tailoring of patient management. These risk assessment tools have the potential to complement and ultimately replace the traditional clinical 10-year risk scores currently used to guide primary preventive clinical management. There is also clear evidence that recent developments in cardiac CT have made it increasingly more feasible for myocardial tissue characterization which previously has been reserved for MRI.^92^

In summary, we are likely to experience quantitative cardiac CT as a comprehensive means for holistic CAD evaluation.
